# Supplementary material for: Losing the Arms Race: Greater Wax Moths Sense but Ignore Bee Alarm Pheromones
Source: Insects. 2019 Mar 23;10(3):81. doi: 10.3390/insects10030081 (PMC6468870; doi:10.3390/insects10030081)
Supplement: Supplementary file 1 [file insects-10-00081-s001.pdf]

Table S1. Statistical results summary of greater wax moth GWM preference or avoidance to bee alarm pheromones in Y maze.

Alarm Compounds	1st Day		3rd Day		5th Day	
	χ^2	p	χ^2	p	χ^2	p
BA	1.679	0.432	20.112	<0.001	2.511	0.285
OA	0.41	0.815	0.366	0.833	4.402	0.111
IPA	0.255	0.88	0.188	0.91	3.489	0.175
2-HP	0.188	0.91	2.145	0.342	5.575	0.062

Table S2. Statistical results summary of GWM preference or avoidance to bee alarm pheromones in oviposition site selection.

Alarm Compounds	Day1		Day2		Sum of Day 1 + Day 2	
	χ^2	p	χ^2	p	χ^2	p
BA	2.45	0.118	0.18	0.671	3.438	0.064
OA	0.501	0.479	0.501	0.479	0.08	0.777
IPA	1.288	0.256	2.02	0.155	1.633	0.201
2-HP	1.633	0.201	1.288	0.256	0.02	0.088
